# Sex‐Specific Genetic Architecture of ALS: Evidence of a Female Protective Effect?

**DOI:** 10.1002/ana.78172

**Published:** 2026-02-10

**Authors:** Maurizio Grassano, Francesca Palumbo, Gabriele Mora, Salvatore Gallone, Giovanni De Marco, Ilaria Merulla, Claudia Paolantonio, Alessandra Maccabeo, Antonio Canosa, Umberto Manera, Rosario Vasta, Barbara Iazzolino, Marcella Testa, Giuseppe Fuda, Paolina Salamone, Giulia Marchese, Federico Casale, Cristina Moglia, Andrea Calvo, Giuseppe Borghero, Adriano Chiò

**Affiliations:** ^1^ Rita Levi Montalcini Department of Neuroscience University of Turin Turin Italy; ^2^ Neurology Unit 1U “City of Health and Science” University Hospital Turin Italy; ^3^ Institute of Neurology Azienda Ospedaliero Universitaria di Cagliari Cagliari Italy; ^4^ Institute of Cognitive Sciences and Technologies National Council of Research Rome Italy

## Abstract

**Background:**

Amyotrophic lateral sclerosis (ALS) shows sex differences in incidence and age of onset, yet the underlying biological mechanisms remain poorly understood.

**Methods:**

We investigated sex‐specific genetic architecture in an Italian ALS cohort with whole‐genome sequencing (1,333 ALS cases, 755 controls). We performed a sex‐stratified burden analysis of rare variants in ALS‐associated genes and compared the proportions of male and female ALS patients carrying pathogenic or rare damaging variants. Key findings were replicated in the AnswerALS cohort (*n* = 723). Gene‐specific sex ratios and familial history for *C9ORF72*, *SOD1*, and *TARDBP* were examined in an expanded dataset of 2,301 Italian ALS patients.

**Results:**

Sex‐stratified burden testing revealed that rare variants in ALS genes were enriched in female cases versus controls (odds ratio [OR] 5.47, 95% confidence interval [CI] 1.60–34.29) but not in male cases. Female ALS patients more frequently carried rare damaging variants compared to males (23.2% vs 18.3%; OR 1.38, 95% CI 1.05–1.81), a finding that was replicated in the AnswerALS cohort (18.9% vs 12.4%; OR 1.58, 95% CI 1.10–2.26). Gene‐level analyses of *TARDBP* carriers revealed a male predominance (2.1:1), yet a higher rate of familial history among females (40.4% vs 24.5%; OR 2.13, 95% CI 1.03–4.39).

**Interpretation:**

Females with ALS exhibited a higher overall burden of rare damaging variants, suggesting sex‐related differences in genetic liability. Gene‐level analyses indicate that the influence of sex varies across ALS genes, particularly *TARDBP*. These findings help explain epidemiological patterns and have implications for the identification of sex‐linked protective mechanisms. ANN NEUROL 2026;99:1536–1544

Amyotrophic lateral sclerosis (ALS) is a devastating neurodegenerative disorder characterized by progressive motor neuron loss, leading to muscle weakness, paralysis, and ultimately death. A notable epidemiological feature of ALS is its sexual dimorphism, with males consistently showing both higher incidence and earlier onset compared to females.^1^, ^2^ Despite these well‐documented sex differences, the biological mechanisms underlying this disparity remain poorly understood.

The “female protective effect” concept has emerged as a compelling framework to explain sex‐based differences in prevalence and clinical presentation across several neurological conditions.^3^ This model suggests that females possess an inherent biological resilience that elevates their threshold for developing disease manifestations. For instance, research in autism spectrum disorders and neurodevelopmental disorders^4^, ^5^ has revealed that affected females typically carry a higher burden of rare de novo deleterious variants compared to affected males, suggesting that females require a greater genetic “hit” to manifest the disorder.

The genetic evidence for a female protective effect in ALS remains largely unexplored. Only a few studies have suggested potential sex‐specific genetic influences in ALS, including differential effects of specific mutations between males and females.^6^

The present study aims to investigate the existence of a female protective effect in ALS through a comprehensive analysis of genetic burden, sex ratios, and familial recurrence in a large cohort of ALS patients and carriers of pathogenic mutations in ALS‐associated genes.

## Methods

### Variant Burden Analysis

#### Study Population

We analyzed whole‐genome sequencing (WGS) data of 1,333 ALS patients (599 females and 734 males) from the Piemonte and Valle d'Aosta Register for ALS (PARALS), an ongoing population‐based register that has systematically collected clinical and genetic data on all ALS cases in the Piedmont region of Italy since 1995.^7^

The control cohort consisted of 755 neurologically healthy individuals (382 females and 373 males) matched to cases by age, ancestry, and geographical origin. Detailed demographic and clinical characteristics for both ALS cases and controls are provided in Table S1.

### WGS

WGS was conducted using standard protocols. Variant calling and annotation were performed using best practices and established pipelines. The detailed sequencing methodology criteria have been previously published.^8^, ^9^ Genome sequencing was not associated with sex, age at onset, disease duration, or family history, minimizing selection bias.

### Sex‐Stratified Case–Control Burden Analysis

To evaluate whether rare genetic variation contributes differently to ALS risk in males and females, we performed a sex‐stratified case–control burden analysis. Rare variants (minor allele frequency [MAF] <0.1% in gnomAD) were aggregated across a panel of genes associated with ALS (https://clinicalgenome.org/). Within each sex, ALS case–control status was modeled using logistic regression, adjusting for age at sampling and the first 5 genome‐wide principal components to account for population stratification. The odds ratios (ORs) from these models reflect the relative enrichment of rare variants across ALS‐associated genes in cases compared to controls, rather than absolute carrier frequencies.

### Rare Damaging Variant Carrier Analysis

To further assess sex‐specific differences in genetic architecture, we compared the proportion of individuals carrying at least 1 (likely) pathogenic or rare damaging variant in ALS‐associated genes between male and female ALS cases. Analyses were performed both including all ALS‐associated genes and after excluding the 3 most frequently mutated genes (*C9ORF72*, *SOD1*, *TARDBP*) as a sensitivity analysis.

Pathogenic and likely pathogenic variants were defined according to standard guidelines.^10^ Rare damaging variants were defined as variants of uncertain significance with a MAF < 0.01% in gnomAD and other population reference datasets and classified as either (i) predicted loss‐of‐function variants (stop‐gain, frameshift, or canonical splice‐site) or (ii) missense variants predicted to be damaging by multiple in silico pathogenicity classifiers. Associations were estimated using logistic regression adjusted for age at disease onset, and reported as odds ratios with 95% confidence intervals (CIs).

### Replication Analysis in AnswerALS Cohort

To validate our findings in an independent population, we performed a replication analysis using data from the AnswerALS consortium, a large‐scale, collaborative research initiative.^11^ We analyzed whole genome sequencing data from 723 ALS patients (264 females, 459 males), excluding asymptomatic carriers of ALS‐related variants and other non‐ALS motor‐neuron diseases. Rare damaging variants were identified and filtered using the same criteria applied to the PARALS cohort. Sex‐specific differences in rare variant burden were assessed using logistic regression, adjusting for age and ethnicity.

### Gene‐Level Analysis

#### Study Population

Following the rare variant burden analysis, we next sought to determine whether sex modifies the effect of specific ALS genes. Because sex‐specific effects are only statistically detectable for genes with sufficient carrier numbers, we restricted our analyses to the 3 most frequent ALS genes in the Italian population—*C9ORF72*, *TARDBP*, and *SOD1*—and assessed whether the distribution of pathogenic/likely pathogenic variants differed between males and females. Other ALS‐associated genes were too rare in our dataset to allow reliable sex‐specific inference.

Gene‐level analyses were performed in a combined cohort of 2,301 ALS patients from 2 Italian regions. The larger group from Piemonte (*n* = 1,850) was derived from the population‐based Piemonte and Aosta Register for ALS (PARALS) and included the 1,333 ALS individuals with WGS data: the clinical characteristics of the overall cohort were similar to the sub‐cohort with WGS data. The Sardinian cohort (*n* = 451) consisted of incident ALS cases of strictly Sardinian ancestry (individuals with both parents of Sardinian origin) identified at regional ALS centers between 2010 and 2019. The unique genetic background of Sardinia, shaped by historical isolation and founder effects, provides a valuable setting for genetic studies.^12^ Demographic and clinical details for both cohorts are provided in Tables S2.

### Genetic Analysis

All 2,301 individuals underwent targeted genetic testing (Sanger sequencing) for variants in all exons of *SOD1* and *TARDBP*. For *C9ORF72*, we used a repeat‐primer polymerase chain reaction (PCR) assay to detect the pathogenic GGGGCC hexanucleotide expansion in the first intron, applying a pathological threshold of ≥30 repeats combined with the characteristic sawtooth pattern.^13^

### Sex Ratio Analysis

To evaluate sex‐specific differences in mutation prevalence, we calculated male‐to‐female ratios among carriers of pathogenic/likely pathogenic in *C9ORF72*, *SOD1*, and *TARDBP*. We used binomial tests to determine whether these ratios significantly deviated from the expected distribution: the null hypothesis assumed equal probability of mutation carriage in males and females (1:1), independent of the overall sex ratio of ALS, allowing detection of sex‐specific deviations in mutation prevalence. Furthermore, we computed ORs and 95% CIs using logistic regression models, incorporating age and cohort of origin as covariates; in these models, female sex was used as the reference category, so that ORs > 1 indicate higher mutation prevalence in males.

To account for potential founder effects, we performed a sensitivity analysis restricted to carriers of the TARDBP p.A382T mutation. This variant is particularly prevalent in Sardinia and has been associated with reduced penetrance.^14^

It is important to note that these gene‐specific analyses address a different question from the case–control rare variant burden analysis. While the burden analysis evaluates sex‐specific differences in genetic susceptibility to ALS, the gene‐level sex ratio analyses assess whether, among individuals with ALS, pathogenic variants in specific genes are differentially distributed between males and females, consistent with sex‐modified penetrance or expressivity.

### Family History Analysis

To explore potential sex‐related differences in familial aggregation, we assessed the frequency of a positive family history of ALS or frontotemporal dementia (FTD) among probands carrying pathogenic/likely pathogenic variants in the 3 major genes. Family history was defined as the presence (yes/no) of at least 1 affected first‐degree relative. This information was routinely collected during standard neurological evaluations or genetic counseling sessions and was available for the entire cohort. Sex differences in family history were evaluated using logistic regression models adjusted for age and cohort of origin; in this model, male sex was used as the reference category, so that ORs >1 indicate higher recurrence in female carriers.

As for the prevalence analysis, we performed a sensitivity analysis restricted to carriers of the *TARDBP p.A382T* founder variant.

### Statistical Analysis

All statistical analyses were performed in R version 4.4.3, with a significance threshold of *p* < 0.05.

### Ethical Approval

All participants provided written informed consent, and the study protocols were approved by the respective regional ethics committees (University Hospital Città della Salute e della Scienza of Torino, 0036344; Comitato Etico della Sardegna, NP/2023/1562).

## Results

### Sex‐Stratified Case–Control Burden Analysis

To determine whether the burden of rare variants in ALS‐associated genes differs between sexes, we first assessed whether the burden of rare damaging variants in ALS‐associated genes differed between sexes using whole‐genome sequencing data from 1,333 ALS patients (599 females, 734 males) and 755 geographically matched controls (382 females, 373 males).

Across all participants, ALS cases showed a significant enrichment of rare variants in ALS‐related genes compared with controls (OR 3.33, 95% CI 1.42–9.80, *p* = 0.0122).

**FIGURE 1:**
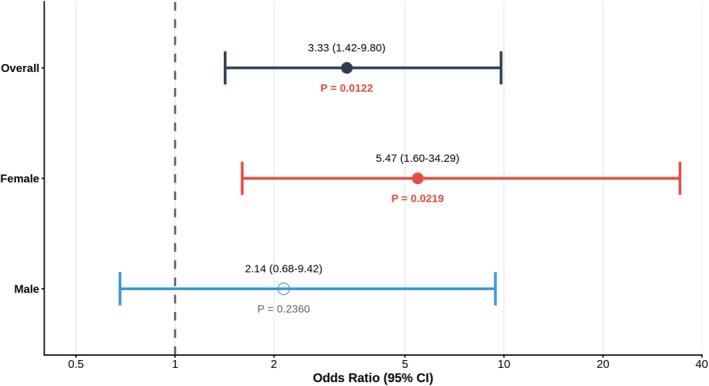
Sex‐stratified case–control burden analysis of rare variants in amyotrophic lateral sclerosis (ALS) ‐associated genes. Forest plot showing odds ratios (ORs) and 95% confidence intervals (CIs) from logistic regression models testing the association between rare variant burden in ALS‐associated genes and ALS status in the Piemonte and Valle d'Aosta Register for ALS (PARALS) cohort (1,333 ALS cases and 755 controls). Analyses were performed in the overall cohort and separately in females and males. Within each stratum, ALS cases were compared with sex‐matched controls. Models were adjusted for age at sampling and the first 5 principal components of ancestry. *p*‐Values correspond to the case–control burden test within each stratum. [Color figure can be viewed at www.annalsofneurology.org]

When stratified by sex, this enrichment was observed only in females. Female ALS cases showed a significant enrichment of rare variants in ALS‐associated genes (OR 5.47, 95% CI 1.60–34.29, *p* = 0.0219), whereas no significant enrichment was detected among males (OR 2.14, 95% CI 0.68–9.42, *p* = 0.2360) (Fig 1).

### Prevalence of Rare Pathogenic Variants

We next compared the prevalence of (likely) pathogenic or rare damaging variant between male and female ALS cases. Among sequenced individuals, 23.2% of females (139/599) and 18.3% of males (134/734) carried at least 1 such variant. This corresponds to 38% higher odds in females (OR 1.38, 95% CI 1.05–1.81, *p* = 0.0112; Table S3).

After removing mutations in these genes from the analysis, 12.9% of female ALS cases still carried rare damaging variants in other ALS‐associated genes, compared to 9.8% of male cases (OR 1.54, 95% CI 1.09–2.18, *p* = 0.0141).

### Replication in the AnswerALS Cohort

**FIGURE 2:**
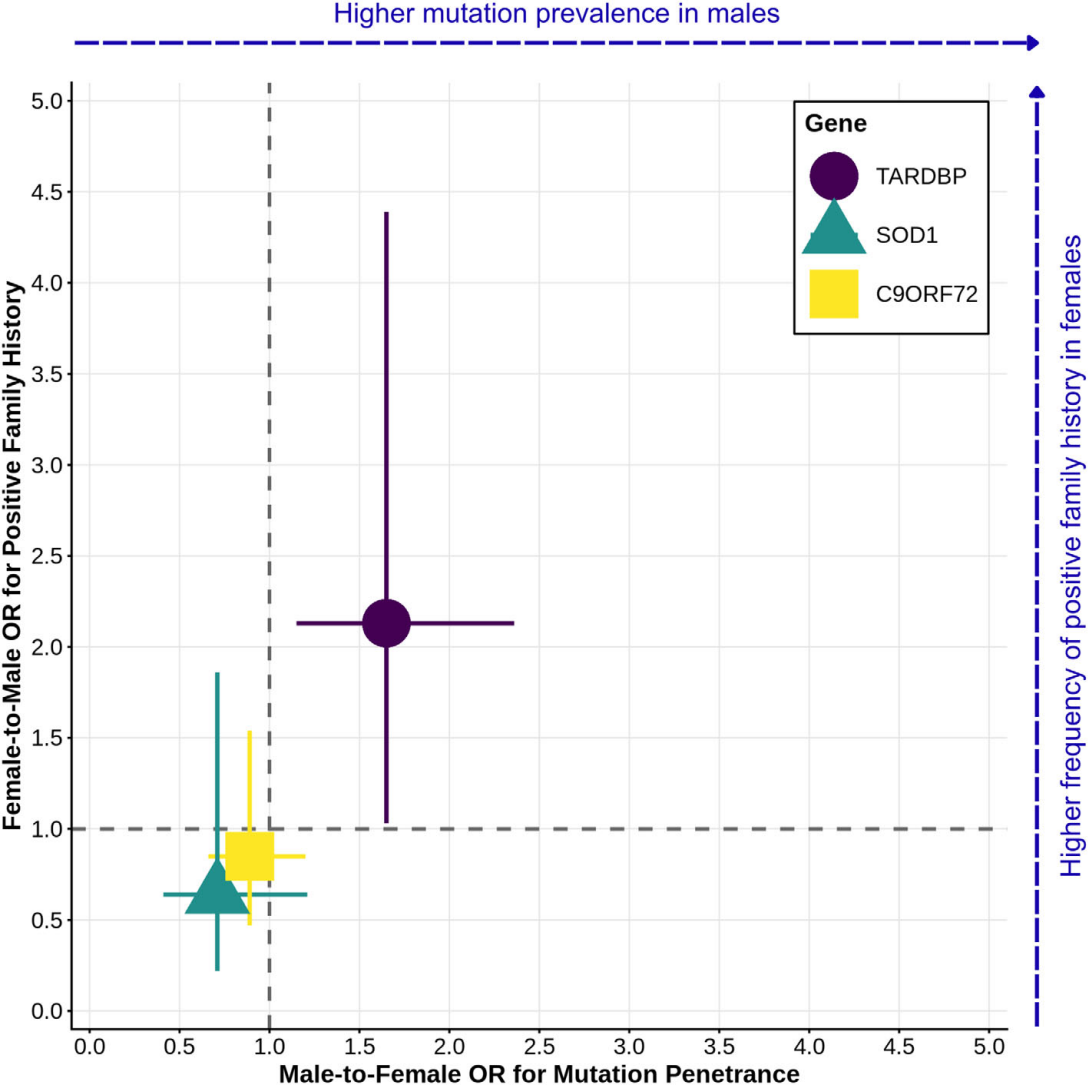
Sex‐specific patterns of variant prevalence and familial history across major amyotrophic lateral sclerosis (ALS) genes sex‐stratified odds ratios for *TARDBP* (*n* = 145 carriers), *SOD1* (*n* = 54), and *C9ORF72* (*n* = 184) in 2,301 Italian ALS patients. The x‐axis shows male‐to‐female odds ratio (OR) for carrier prevalence (values > 1 indicate male excess). The y‐axis shows female‐to‐male OR for positive family history among carriers (values > 1 indicate higher familial recurrence in females). Error bars represent 95% confidence intervals; ORs with confidence intervals excluding 1.0 are statistically significant. *TARDBP* exhibits both male‐biased carrier prevalence and elevated familial recurrence in female carriers, consistent with a higher liability threshold for females. *C9ORF72* and *SOD1* show no significant sex differences in either dimension. [Color figure can be viewed at www.annalsofneurology.org]

To validate our findings in an independent cohort, we analyzed whole‐genome data of 723 ALS patients (264 females, 459 males) from the AnswerALS consortium. In line with our primary analysis, we observed a significantly higher frequency of ALS‐related variants in female ALS patients compared to males (Table S4). Among female ALS cases, 18.9% (50/264) carried at least 1 pathogenic or rare damaging variant in an ALS‐associated gene, compared to 12.4% (57/459) of male patients. Females showed higher odds of carrying ALS‐related variants compared to males (OR 1.58, 95% CI 1.10–2.26, *p* = 0.0128) (Fig 2). However, no difference was observed when removing pathogenic variants in C9ORF72, SOD1 and TARDBP (OR 1.20, 95% CI 0.79–1.96, *p* = 0.6120).

### Gene‐Specific Sex Differences in Prevalence and Familial Recurrence

Having established overall differences in variant burden, we next examined whether specific genes showed sex‐specific patterns. We analyzed 2,301 ALS patients from 2 Italian regions: 451 patients from Sardinia (195 females, 43.2%) and 1,850 patients from PARALS (834 females, 45.1%). The combined cohort consisted of 1,029 females (44.7%) and 1,272 males (55.3%). Because these analyses address sex differences among affected individuals rather than disease susceptibility, odds ratios in the following gene‐specific analyses quantify differences in mutation prevalence between male and female ALS cases.

We identified 145 carriers of pathogenic or likely pathogenic variants in TARDBP (6.1% of the total cohort), a frequency in line with previous studies in this population.^14^ *TARDBP* variants showed a marked male predominance: the male‐to‐female ratio among carriers was approximately 2.1:1 (98 males, 47 females), significantly deviating from the expected distribution (binomial test *p* < 0.0001). This sex imbalance was also observed when restricting analyses to the *TARDBP* p.A382T founder mutation (82 males, 40 females, male‐to‐female ratio 2.1:1, *p* = 0.00018) and was similarly observed within both the PARALS (39 males, 23 females; male‐to female ratio 1.7:1; *p* = 0.0559) and Sardinia (59 males, 24 females; male‐to‐female ratio 2.5:1; *p* = 0.0011) cohorts (Table 1).

**TABLE 1:** Sex‐Specific Distribution of Pathogenic Variants in Major ALS Genes

Parameter	Total Cohort (N = 2,301)	*p*‐Value
Sex, *n* (%)		
Female	1,029 (44.7%)	
Male	1,272 (55.3%)	
Age at onset, mean (SD)		
Female	65.1 (11.4)	‐
Male	64.1 (11.5)	0.0281
All pathogenic variant carriers	393	
Female	165 (16.0%)	‐
Male	228 (17.9%)	‐
*TARDBP* pathogenic variant carriers		
Female	47 (4.6%)	‐
Male	98 (7.7%)	‐
Male‐to‐female ratio	2.1:1	<0.0001
*TARDBP* p.A382T carriers		
Female	40 (3.9%)	‐
Male	82 (6.4%)	‐
Male‐to‐female ratio	2.1:1	0.0002
*C9ORF72* GGGCC expansion carriers		
Female	85 (8.3%)	‐
Male	99 (7.9%)	‐
Male‐to‐female ratio	1.2:1	0.2804
*SOD1* pathogenic variant carriers		
Female	28 (2.7%)	‐
Male	26 (2.0%)	‐
Male‐to‐female ratio	0.9:1	0.7495

*Note:* Combined cohort of 2,301 Italian ALS patients (Piemonte and Valle d'Aosta Register for ALS [PARALS] and Sardinian cohorts). Carrier frequencies are presented as n (% of sex‐specific total). Male‐to‐female ratios were tested against the expected 1:1 distribution using binomial tests. For TARDBP, analyses are shown for all pathogenic variants and separately for the p.A382T founder mutation. Age at onset compared between sexes using Student's *t*‐test. Cohort‐specific data are reported in Table S3.

Overall, males had 65% higher odds of carrying a *TARDBP* mutation compared to females among ALS cases (OR 1.65, 95% CI 1.15–2.36, P = 0.0061). In contrast, no significant sex differences were observed in the frequency of mutations in either C9ORF72 (99 males, 85 females; OR 0.89, 95% CI 0.66–1.20, *p* = 0.435) or SOD1 (26 males, 28 females; OR 0.71, 95% CI 0.41–1.21, *p* = 0.210), despite a numerically lower male‐to‐female ratio among SOD1 carriers compared with the overall ALS sex ratio (Table 1).

Despite their lower mutation frequency, female *TARDBP* carriers showed significantly higher rates of ALS history among their first‐degree relatives. Affected females with a *TARDBP* mutation displayed a 40.4% (19/47) recurrence rate, substantially higher than the 24.5% (24/98) observed in affected males. This pattern persisted when restricting the analysis to *TARDBP* p.A382T carriers (41.0% in affected mothers vs. 25.3% in affected fathers).

After adjusting for age and cohort, female TARDBP carriers more frequently reported a positive family history of ALS compared to male carriers (OR 2.13, 95% CI 1.03–4.39, *p* = 0.041) (Fig 2). This sex‐specific recurrence pattern was not observed for the highly penetrant mutations in *SOD1* (46.4% in females vs 57.7% in males; OR 0.64, 95% CI 0.22–1.86, *p* = 0.430) or *C9ORF72* (58.8% in females vs 62.6% in males; OR 0.85, 95% CI 0.47–1.54, *p* = 0.651).

## Discussion

In this study, we identified several lines of evidence supporting sex‐specific differences in the genetic architecture of ALS. Across 2 independent cohorts, female ALS patients carried a consistently higher burden of variants in ALS‐associated genes. This robust, cross‐cohort finding aligns with established epidemiological observations in ALS and suggests that females may have a higher liability threshold, requiring a greater cumulative genetic load to develop disease—a pattern potentially compatible with a female protective mechanism. Alternatively, and not mutually exclusive, these findings may indicate that genetic factors contribute more strongly to ALS risk in females relative to non‐genetic or environmental influences.

Within this broader context, sex differences were not uniform across genes. We observed a male predominance among carriers of pathogenic variants in *TARDBP*, particularly the *TARDBP* p.A382T founder variant, whereas the prevalence of pathogenic variants in *C9ORF72* and *SOD1* did not differ between males and females. These findings confirm earlier observations from studies^15^ on a smaller cohort from literature data, suggesting that *TARDBP* may be uniquely sensitive to sex‐modified penetrance and that that gene‐specific sex differences may be population‐dependent, and possibly influenced by local founder effects or mutation frequencies. One alternative explanation for the observed male predominance among *TARDBP* mutation carriers is a sex difference in background allele frequency. However, *TARDBP* is an autosomal gene, and there is no a priori expectation that pathogenic variants such as p.A382T would be more prevalent in males than females in the general population.

Female *TARDBP* carriers also exhibited higher rates of familial history of ALS compared to male carriers, aligning with predictions of the Carter effect, wherein the less frequently affected sex (here, females) must exceed a higher liability threshold to manifest disease and consequently transmits greater genetic risk to offspring (Fig 2). *TARDBP* may exhibit incomplete penetrance that is further modified by sex‐specific factors, with males developing disease at lower genetic thresholds. Alternatively, ascertainment bias could play a role if male *TARDBP* carriers with sporadic presentations are more readily diagnosed.

The presence of gene‐specific pathways suggests that sex may interact differently with distinct pathogenic mechanisms in ALS. One possible explanation is that sex‐related protective factors have a greater impact on variants with intermediate or variable penetrance,^16^, ^17^ whereas the strong pathogenic effects of highly penetrant mutations (e.g., *SOD1*, *C9ORF72*) may diminish the observable influence of sex. However, this remains speculative and must be interpreted with caution, particularly given the population‐specific distribution of *TARDBP* variants in Italy.^18^

Several biological mechanisms may account for the heightened genetic resilience observed in female ALS patients. Central to this sex‐based difference are sex hormones,^19^ particularly estrogen, with estrogen exhibiting a range of protective effects.^20^ Additionally, sex‐specific transcriptional landscapes^21^ and gene expression patterns^22^ could play a significant role. The immune system also shows sexual dimorphism that is pertinent to ALS pathogenesis.^23^, ^24^, ^25^, ^26^ Furthermore, the higher levels of TDP‐43 found in the cerebrospinal fluid of males with ALS^27^ suggest sex‐specific differences in TDP‐43 pathology or clearance mechanisms, which may be particularly relevant to our findings.

Our study has several limitations that warrant consideration. First, replication in additional populations with different ancestral backgrounds is necessary to establish the generalizability of our findings. While the overall sex difference in pathogenic variant burden was validated in an independent multi‐ancestry cohort, the gene‐specific patterns require further validation in non‐Italian populations. However, validation of the strong male predominance among *TARDBP* carriers may be challenging, as the p.A382T founder mutation is rarer outside Italy.

An important methodological limitation concerns our assessment of familial penetrance. Ideally, we would have calculated these rates using complete family trees to directly compare disease transmission likelihoods from affected mothers versus affected fathers. Due to dataset limitations, we used familial history of disease as a proxy measure. This approach, while informative, may not capture the full complexity of inheritance patterns and could introduce bias if systematic differences exist in family size or structure between male and female probands. Future studies leveraging comprehensive genealogical data and prospective family‐based designs will be needed to accurately quantify sex‐specific penetrance.

Finally, the contribution of sex‐specific effects on common polygenic risk remains unexplored, as our cohorts were underpowered to investigate whether sex‐specific pathways existed beyond rare variants of large effect.

Although our findings are compatible with a female protective model, alternative explanations must be acknowledged. Recent research in autism spectrum disorders has emphasized that the liability threshold model may not fully encapsulate the complexities of sex differences in neurological disorders.^28^ Other potential mechanisms include distinct pathogenic pathways or environmental and lifestyle factors that could interact with genetic risk. Our study cannot conclusively rule out these alternative explanations. Future research integrating genomic, transcriptomic, hormonal, and other molecular data will be necessary to evaluate different models and explain sex differences in ALS.

Taken together, our results highlight the importance of considering sex as a key variable when interpreting genetic risk and biological mechanisms involved in ALS pathogenesis. This also underscores the importance of considering both specific genetic causes and sex when assessing disease risk and prognosis in ALS patients.^29^ The stronger association between rare genetic burden and ALS in females, along with the gene‐specific effects observed in *TARDBP*, suggests a complex interplay between genetic architecture and sex‐related biological mechanisms. If confirmed, these findings may eventually inform sex‐specific risk prediction, genetic counselling, and therapeutic development. Understanding why females appear more resilient to ALS could provide novel insights into disease pathogenesis and identify protective pathways with clinical relevance.

## Author Contributions

M.G., F.P., G.Mo., G.B., A.Ch. contributed to the conception and design of the study. M.G., F.P., G.Mo., C.M., G.B., A.Cal., A.Ch. contributed to drafting the text or preparing the figures. M.G., F.P., G.Mo., S.G., G.D.M., I.M., C.P., A.M., A.Can., U.M., R.V., B.I., M.T., G.F., P.S., G.Ma., F.C., C.M., G.B., A.Cal., A.Ch. contributed to the acquisition and analysis of data.

## Potential Conflicts of Interests

The authors report no competing interests relevant to this work.

## Supporting information


**Table S1.** Sex‐Stratified Demographics and Rare Variant Burden in PARALS and AnswerALS Cohorts.
**Table S2.** Sex‐Specific Carrier Frequencies and Male‐to‐Female Ratios for Major ALS Genes in PARALS and Sardinian Cohorts.
**Table S3.** Complete List of Pathogenic, Likely Pathogenic, and Rare Damaging Variants Identified by Whole‐Genome Sequencing in the PARALS Cohort.
**Table S4.** Complete List of Pathogenic, Likely Pathogenic, and Rare Damaging Variants Identified by Whole‐Genome Sequencing in the AnswerALS Cohort.

## Data Availability

Qualified investigators with suitable research projects may request the data from the senior authors. Data used in the preparation of this article were also obtained from the Answer ALS Data Portal (AALS‐01184). For up‐to‐date information on the study, visit https://dataportal.answerals.org.
