# Supplementary material for: Sex‐Specific Genetic Architecture of ALS: Evidence of a Female Protective Effect?
Source: Ann Neurol. 2026 Feb 10;99(6):1536–44. doi: 10.1002/ana.78172 (PMC13206350; doi:10.1002/ana.78172)
Supplement: Supplementary file 1 — Table S1. Sex‐Stratified Demographics and Rare Variant Burden in PARALS and AnswerALS Cohorts. Table S2. Sex‐Specific Carrier Frequencies and Male‐to‐Female Ratios for Major ALS Genes in PARALS and Sardinian Cohorts. Table S3. Complete List of Pathogenic, Likely Pathogenic, and Rare Damaging Variants Identified by Whole‐Genome Sequencing in the PARALS Cohort. Table S4. Complete List of Pathogenic, Likely Pathogenic, and Rare Damaging Variants Identified by Whole‐Genome Sequencing in the AnswerALS Cohort. [file ANA-99-1536-s001.docx]

**Supplementary Files**

| **Table S1.** Sex-Stratified Demographics and Rare Variant Burden in PARALS and AnswerALS Cohorts | **2** |
| --- | --- |
| **Table S2.** Sex-Specific Carrier Frequencies and Male-to-Female Ratios for Major ALS Genes in PARALS and Sardinian Cohorts | **3** |
| **Table S3.** Complete List of Pathogenic, Likely Pathogenic, and Rare Damaging Variants Identified by Whole-Genome Sequencing in the PARALS Cohort | **4** |
| **Table S4.** Complete List of Pathogenic, Likely Pathogenic, and Rare Damaging Variants Identified by Whole-Genome Sequencing in the AnswerALS Cohort | **11** |

**Table S1.** Sex-Stratified Demographics and Rare Variant Burden in PARALS and AnswerALS Cohorts

|  | **PARALS (N=1,333)** | **AnswerALS (N =723)** |
| --- | --- | --- |
| **Sex** |  |  |
| Female | 599 (44.9%) | 264 (36.5%) |
| Male | 734 (55.1%) | 459 (63.5%) |
| **Age at onset, mean (SD)** | 66.2 (11.0) | 56.2 (11.7) |
| Female | 66.6 (11.2) | 57.4 (12.5) |
| Male | 65.8 (10.8) | 55.6 (11.2) |
| **Site of onset: Bulbar** | 418 (32.2%) | 187 (25.9%) |
| Female | 246 (41.1%) | 90 (34.1%) |
| Male | 172 (23.4%) | 97 (21.1%) |
| **ALSFRS-R at baseline, mean (SD)** | 40.0 (6.8) | 34.1 (8.5) |
| Female | 39.9 (6.7) | 34.5 (8.5) |
| Male | 40.1 (6.9) | 33.2 (8.4) |
| **All variant carriers** | **273 (20.5%)** | **107 (14.8%)** |
| Female | 139 (23.2%) | 50 (18.9%) |
| Male | 134 (18.2%) | 57 (12.4%) |
| ***C9ORF72* GGGGCC expansion carriers** | **92 (6.9%)** | **42 (5.8%)** |
| Female | 45 (7.5%) | 24 (9.1%) |
| Male | 47 (6.4%) | 18 (3.9%) |
| ***SOD1* variant carriers** | **18 (1.4%)** | **19 (2.6%)** |
| Female | 12 (2.0%) | 8 (3.0%) |
| Male | 6 (0.8%) | 11 (2.4%) |
| ***TARDBP* variant carriers** | **14 (1.1%)** | **6 (0.8%)** |
| Female | 5 (0.8%) | 2 (0.8%) |
| Male | 9 (1.2%) | 4 (0.9%) |
| **Other variant carriers** | **149 (11.2%)** | **40 (5.5%)** |
| Female | 77 (12.9%) | 16 (6.0%) |
| Male | 72 (9.8%) | 24 (5.2%) |

**Table S2.** Sex-Specific Carrier Frequencies and Male-to-Female Ratios for Major ALS Genes in PARALS and Sardinian Cohorts.

|  | **PARALS (N = 1,850)** | **Sardinia (N = 451)** |
| --- | --- | --- |
| **Sex, n (%)** |  |  |
| Female | 834 (45.1%) | 195 (43.2%) |
| Male | 1,016 (54.9%) | 256 (56.8%) |
| **Age at onset, mean (SD)** | 66.1 (11.0) | 61.2 (11.7) |
| Female | 66.6 (11.3) | 62.0 (12.2) |
| Male | 64.8 (11.4) | 60.6 (11.2) |
| **Site of onset: Bulbar, n (%)** | 590 | 107 |
| Female | 334 | 52 |
| Male | 256 | 55 |
| **Other mutation carriers, n (%)** | **279 (15.1%)** | **114 (25.3%)** |
| Female | 134 (16.1%) | 31 (15.9%) |
| Male | 145 (14.3%) | 83 (32.4%) |
| ***TARDBP* mutation carriers, n (%)** | **62 (3.4%)** | **83 (18.4%)** |
| Female | 23 (2.8%) | 24 (12.3%) |
| Male | 39 (3.8%) | 59 (23.0%) |
| Male-to-female ratio | 1.7:1 | 2.5:1 |
| ***TARDBP* p.A382T carriers, n (%)** | **47 (2.5%)** | **73 (16.2)** |
| Female | 20 (2.4%) | 18 (9.2%) |
| Male | 27 (2.7%) | 55 (21.5%) |
| Male-to-female ratio | 1.4:1 | 3.1:1 |
| ***C9ORF72* carriers, n (%)** | **157 (8.5%)** | **27 (6.0%)** |
| Female | 78 (9.4%) | 7 (3.6%) |
| Male | 79 (7.8%) | 20 (7.8%) |
| Male-to-female ratio | 1.0:1 | 2.9:1 |
| ***SOD1* carriers, n (%)** | **50 (2.7%)** | **4 (0.9%)** |
| Female | 28 (3.4%) | 0 (0.0%) |
| Male | 22 (2.2%) | 4 (1.6%) |
| Male-to-female ratio | 0.8:1 | - |

**Table S3.** Complete List of Pathogenic, Likely Pathogenic, and Rare Damaging Variants Identified by Whole-Genome Sequencing in the PARALS Cohort

| **Gene** | **Exon** | **Nucleotide change** | **Amino acid change** | **Sex** | **Age at ALS onset** |
| --- | --- | --- | --- | --- | --- |
| ***ANXA11*** | 3 | c.A119G | p.D40G | Female | 55.3 |
| ***ANXA11*** | 3 | c.G102A | p.M34I | Male | 60.3 |
| ***ANXA11*** | 6 | c.C731T | p.T244M | Male | 63.3 |
| ***ANXA11*** | 3 | c.A119G | p.D40G | Female | 61.9 |
| ***ANXA11*** | 8 | c.G905A | p.R302H | Female | 76.3 |
| ***ANXA11*** | 9 | c.T1010A | p.L337H | Female | 77.9 |
| ***ANXA11*** | 4 | c.C173T | p.A58V | Male | 78.1 |
| ***ANXA11*** | 6 | c.C731T | p.T244M | Female | 66.6 |
| ***ANXA11*** | 12 | c.G1191C | p.E397D | Female | 45.2 |
| ***ANXA11*** | 8 | c.C922T | p.R308X | Male | 77.6 |
| ***ANXA11*** | 3 | c.G102A | p.M34I | Male | 69.2 |
| ***ANXA11*** | 2 | c.T7C | p.Y3H | Female | 75.5 |
| ***C9ORF72*** | intron1 | c.-45+163GGGGCC[>24] | - | Male | 45.3 |
| ***C9ORF72*** | intron1 | c.-45+163GGGGCC[>24] | - | Male | 77.4 |
| ***C9ORF72*** | intron1 | c.-45+163GGGGCC[>24] | - | Female | 56.3 |
| ***C9ORF72*** | intron1 | c.-45+163GGGGCC[>24] | - | Male | 71.0 |
| ***C9ORF72*** | intron1 | c.-45+163GGGGCC[>24] | - | Male | 45.3 |
| ***C9ORF72*** | intron1 | c.-45+163GGGGCC[>24] | - | Male | 71.3 |
| ***C9ORF72*** | intron1 | c.-45+163GGGGCC[>24] | - | Female | 59.8 |
| ***C9ORF72*** | intron1 | c.-45+163GGGGCC[>24] | - | Male | 69.5 |
| ***C9ORF72*** | intron1 | c.-45+163GGGGCC[>24] | - | Female | 74.7 |
| ***C9ORF72*** | intron1 | c.-45+163GGGGCC[>24] | - | Female | 58.1 |
| ***C9ORF72*** | intron1 | c.-45+163GGGGCC[>24] | - | Female | 60.2 |
| ***C9ORF72*** | intron1 | c.-45+163GGGGCC[>24] | - | Female | 72.4 |
| ***C9ORF72*** | intron1 | c.-45+163GGGGCC[>24] | - | Male | 56.8 |
| ***C9ORF72*** | intron1 | c.-45+163GGGGCC[>24] | - | Male | 63.2 |
| ***C9ORF72*** | intron1 | c.-45+163GGGGCC[>24] | - | Male | 60.6 |
| ***C9ORF72*** | intron1 | c.-45+163GGGGCC[>24] | - | Male | 61.3 |
| ***C9ORF72*** | intron1 | c.-45+163GGGGCC[>24] | - | Female | 69.1 |
| ***C9ORF72*** | intron1 | c.-45+163GGGGCC[>24] | - | Male | 50.8 |
| ***C9ORF72*** | intron1 | c.-45+163GGGGCC[>24] | - | Male | 74.8 |
| ***C9ORF72*** | intron1 | c.-45+163GGGGCC[>24] | - | Male | 52.1 |
| ***C9ORF72*** | intron1 | c.-45+163GGGGCC[>24] | - | Female | 67.3 |
| ***C9ORF72*** | intron1 | c.-45+163GGGGCC[>24] | - | Female | 67.3 |
| ***C9ORF72*** | intron1 | c.-45+163GGGGCC[>24] | - | Female | 44.5 |
| ***C9ORF72*** | intron1 | c.-45+163GGGGCC[>24] | - | Male | 61.7 |
| ***C9ORF72*** | intron1 | c.-45+163GGGGCC[>24] | - | Female | 48.1 |
| ***C9ORF72*** | intron1 | c.-45+163GGGGCC[>24] | - | Male | 69.8 |
| ***C9ORF72*** | intron1 | c.-45+163GGGGCC[>24] | - | Male | 67.3 |
| ***C9ORF72*** | intron1 | c.-45+163GGGGCC[>24] | - | Male | 65.5 |
| ***C9ORF72*** | intron1 | c.-45+163GGGGCC[>24] | - | Female | 55.7 |
| ***C9ORF72*** | intron1 | c.-45+163GGGGCC[>24] | - | Male | 55.8 |
| ***C9ORF72*** | intron1 | c.-45+163GGGGCC[>24] | - | Male | 64.4 |
| ***C9ORF72*** | intron1 | c.-45+163GGGGCC[>24] | - | Female | 66.7 |
| ***C9ORF72*** | intron1 | c.-45+163GGGGCC[>24] | - | Male | 66.4 |
| ***C9ORF72*** | intron1 | c.-45+163GGGGCC[>24] | - | Female | 70.2 |
| ***C9ORF72*** | intron1 | c.-45+163GGGGCC[>24] | - | Female | 63.8 |
| ***C9ORF72*** | intron1 | c.-45+163GGGGCC[>24] | - | Female | 49.7 |
| ***C9ORF72*** | intron1 | c.-45+163GGGGCC[>24] | - | Male | 59.3 |
| ***C9ORF72*** | intron1 | c.-45+163GGGGCC[>24] | - | Male | 50.5 |
| ***C9ORF72*** | intron1 | c.-45+163GGGGCC[>24] | - | Male | 63.6 |
| ***C9ORF72*** | intron1 | c.-45+163GGGGCC[>24] | - | Male | 64.4 |
| ***C9ORF72*** | intron1 | c.-45+163GGGGCC[>24] | - | Female | 63.9 |
| ***C9ORF72*** | intron1 | c.-45+163GGGGCC[>24] | - | Female | 45.1 |
| ***C9ORF72*** | intron1 | c.-45+163GGGGCC[>24] | - | Female | 77.9 |
| ***C9ORF72*** | intron1 | c.-45+163GGGGCC[>24] | - | Female | 44.4 |
| ***C9ORF72*** | intron1 | c.-45+163GGGGCC[>24] | - | Female | 69.3 |
| ***C9ORF72*** | intron1 | c.-45+163GGGGCC[>24] | - | Male | 54.0 |
| ***C9ORF72*** | intron1 | c.-45+163GGGGCC[>24] | - | Female | 57.5 |
| ***C9ORF72*** | intron1 | c.-45+163GGGGCC[>24] | - | Female | 43.7 |
| ***C9ORF72*** | intron1 | c.-45+163GGGGCC[>24] | - | Male | 44.1 |
| ***C9ORF72*** | intron1 | c.-45+163GGGGCC[>24] | - | Male | 66.2 |
| ***C9ORF72*** | intron1 | c.-45+163GGGGCC[>24] | - | Male | 55.1 |
| ***C9ORF72*** | intron1 | c.-45+163GGGGCC[>24] | - | Male | 71.0 |
| ***C9ORF72*** | intron1 | c.-45+163GGGGCC[>24] | - | Female | 65.8 |
| ***C9ORF72*** | intron1 | c.-45+163GGGGCC[>24] | - | Male | 60.5 |
| ***C9ORF72*** | intron1 | c.-45+163GGGGCC[>24] | - | Female | 58.9 |
| ***C9ORF72*** | intron1 | c.-45+163GGGGCC[>24] | - | Female | 54.7 |
| ***C9ORF72*** | intron1 | c.-45+163GGGGCC[>24] | - | Female | 56.5 |
| ***C9ORF72*** | intron1 | c.-45+163GGGGCC[>24] | - | Female | 64.8 |
| ***C9ORF72*** | intron1 | c.-45+163GGGGCC[>24] | - | Female | 52.8 |
| ***C9ORF72*** | intron1 | c.-45+163GGGGCC[>24] | - | Male | 52.8 |
| ***C9ORF72*** | intron1 | c.-45+163GGGGCC[>24] | - | Female | 72.4 |
| ***C9ORF72*** | intron1 | c.-45+163GGGGCC[>24] | - | Female | 49.7 |
| ***C9ORF72*** | intron1 | c.-45+163GGGGCC[>24] | - | Female | 40.3 |
| ***C9ORF72*** | intron1 | c.-45+163GGGGCC[>24] | - | Female | 44.1 |
| ***C9ORF72*** | intron1 | c.-45+163GGGGCC[>24] | - | Male | 64.3 |
| ***C9ORF72*** | intron1 | c.-45+163GGGGCC[>24] | - | Male | 58.7 |
| ***C9ORF72*** | intron1 | c.-45+163GGGGCC[>24] | - | Male | 70.5 |
| ***C9ORF72*** | intron1 | c.-45+163GGGGCC[>24] | - | Female | 60.4 |
| ***C9ORF72*** | intron1 | c.-45+163GGGGCC[>24] | - | Male | 48.9 |
| ***C9ORF72*** | intron1 | c.-45+163GGGGCC[>24] | - | Female | 74.8 |
| ***C9ORF72*** | intron1 | c.-45+163GGGGCC[>24] | - | Male | 69.8 |
| ***C9ORF72*** | intron1 | c.-45+163GGGGCC[>24] | - | Male | 63.3 |
| ***C9ORF72*** | intron1 | c.-45+163GGGGCC[>24] | - | Male | 66.2 |
| ***C9ORF72*** | intron1 | c.-45+163GGGGCC[>24] | - | Male | 48.1 |
| ***C9ORF72*** | intron1 | c.-45+163GGGGCC[>24] | - | Female | 67.2 |
| ***C9ORF72*** | intron1 | c.-45+163GGGGCC[>24] | - | Male | 58.1 |
| ***C9ORF72*** | intron1 | c.-45+163GGGGCC[>24] | - | Female | 51.3 |
| ***C9ORF72*** | intron1 | c.-45+163GGGGCC[>24] | - | Female | 60.7 |
| ***C9ORF72*** | intron1 | c.-45+163GGGGCC[>24] | - | Male | 51.4 |
| ***C9ORF72*** | intron1 | c.-45+163GGGGCC[>24] | - | Female | 56.8 |
| ***C9ORF72*** | intron1 | c.-45+163GGGGCC[>24] | - | Male | 67.5 |
| ***C9ORF72*** | intron1 | c.-45+163GGGGCC[>24] | - | Male | 39.5 |
| ***C9ORF72*** | intron1 | c.-45+163GGGGCC[>24] | - | Male | 59.1 |
| ***C9ORF72*** | intron1 | c.-45+163GGGGCC[>24] | - | Female | 72.9 |
| ***C9ORF72*** | intron1 | c.-45+163GGGGCC[>24] | - | Female | 54.6 |
| ***C9ORF72*** | intron1 | c.-45+163GGGGCC[>24] | - | Male | 68.9 |
| ***C9ORF72*** | intron1 | c.-45+163GGGGCC[>24] | - | Male | 50.4 |
| ***C9ORF72*** | intron1 | c.-45+163GGGGCC[>24] | - | Male | 56.7 |
| ***C9ORF72*** | intron1 | c.-45+163GGGGCC[>24] | - | Female | 68.5 |
| ***C9ORF72*** | intron1 | c.-45+163GGGGCC[>24] | - | Female | 60.3 |
| ***C9ORF72*** | intron1 | c.-45+163GGGGCC[>24] | - | Female | 54.3 |
| ***C9ORF72*** | intron1 | c.-45+163GGGGCC[>24] | - | Female | 61.9 |
| ***CHCHD10*** | 2 | c.C239T | p.P80L | Female | 70.7 |
| ***CHCHD10*** | 2 | c.C104A | p.A35D | Male | 61.8 |
| ***CHCHD10*** | 2 | c.C239T | p.P80L | Male | 83.7 |
| ***CHCHD10*** | 2 | c.C239T | p.P80L | Female | 55.7 |
| ***CHCHD10*** | 2 | c.C239T | p.P80L | Female | 77.8 |
| ***CHMP2B*** | 2 | c.A85G | p.I29V | Female | 69.9 |
| ***CHMP2B*** | 2 | c.A85G | p.I29V | Female | 37.0 |
| ***CHMP2B*** | 2 | c.A74G | p.Q25R | Female | 65.0 |
| ***CHMP2B*** | 2 | c.A85G | p.I29V | Male | 75.2 |
| ***DCTN1*** | 12 | c.G1534T | p.A512S | Female | 70.2 |
| ***DCTN1*** | 16 | c.C2030G | p.P677R | Female | 66.9 |
| ***DCTN1*** | 6 | c.C659T | p.A220V | Male | 66.1 |
| ***DCTN1*** | 2 | c.C59T | p.A20V | Male | 48.8 |
| ***DCTN1*** | 15 | c.C1856T | p.T619M | Male | 69.8 |
| ***DCTN1*** | 26 | c.G3283A | p.A1095T | Female | 67.8 |
| ***DCTN1*** | 16 | c.T1937C | p.I646T | Male | 60.3 |
| ***DCTN1*** | 10 | c.G1193C | p.R398P | Male | 77.1 |
| ***DCTN1*** | 18 | c.A2231G | p.Y744C | Male | 70.5 |
| ***DCTN1*** | 26 | c.C3284T | p.A1095V | Male | 57.8 |
| ***DCTN1*** | 18 | c.T2297C | p.M766T | Female | 80.0 |
| ***DCTN1*** | 6 | c.C673T | p.Q225X | Male | 73.8 |
| ***DCTN1*** | 3 | c.A352G | p.K118E | Male | 34.0 |
| ***DCTN1*** | 5 | c.T593C | p.V198A | Female | 60.4 |
| ***FUS*** | 14 | c.C1480T | p.R494X | Female | 28.8 |
| ***FUS*** | 15 | c.G1539C | p.R513S | Female | 48.1 |
| ***FUS*** | 2 | c.A14G | p.D5G | Female | 74.8 |
| ***FUS*** | 15 | c.C1552G | p.Q518E | Female | 51.8 |
| ***FUS*** | 14 | c.C1480T | p.R494X | Female | 66.1 |
| ***GRN*** | 8 | c.A712C | p.T238P | Female | 75.4 |
| ***GRN*** | 3 | c.C203T | p.S68F | Male | 77.0 |
| ***GRN*** | 2 | c.A52G | p.T18A | Male | 36.8 |
| ***GRN*** | 4 | c.G277A | p.G93R | Male | 79.8 |
| ***GRN*** | 8 | c.G777C | p.K259N | Male | 75.6 |
| ***HNRNPA1*** | 8 | c.G824T | p.G275V | Female | 71.4 |
| ***HNRNPA1*** | 6 | c.C666G | p.F222L | Male | 57.3 |
| ***HNRNPA1*** | 8 | c.G883A | p.G295R | Male | 56.6 |
| ***HNRNPA1*** | 8 | c.C876G | p.N292K | Male | 54.1 |
| ***HNRNPA1*** | 4 | c.A380G | p.Q127R | Female | 41.8 |
| ***KIF5A*** | 11 | c.T1196G | p.L399R | Female | 74.7 |
| ***KIF5A*** | 11 | c.G1279A | p.V427M | Female | 57.5 |
| ***KIF5A*** | 16 | c.C1867T | p.R623W | Female | 78.3 |
| ***KIF5A*** | 24 | c.A2750G | p.N917S | Female | 46.8 |
| ***KIF5A*** | 17 | c.C2033T | p.T678I | Female | 71.7 |
| ***KIF5A*** | 17 | c.G1996A | p.E666K | Female | 63.2 |
| ***KIF5A*** | 13 | c.G1600A | p.G534R | Male | 60.1 |
| ***KIF5A*** | 4 | c.C340T | p.R114X | Male | 68.7 |
| ***KIF5A*** | 22 | c.2489delC | p.K831Nfs*128 | Female | 48.4 |
| ***KIF5A*** | 6 | c.G475T | p.V159L | Female | 74.9 |
| ***KIF5A*** | 16 | c.C1885T | p.R629W | Male | 64.0 |
| ***MATR3*** | 2 | c.A368G | p.D123G | Male | 63.2 |
| ***MATR3*** | 12 | c.G1420A | p.A474T | Female | 62.7 |
| ***MATR3*** | 11 | c.C1256T | p.S419L | Female | 83.6 |
| ***MATR3*** | 11 | c.G1264A | p.A422T | Male | 75.7 |
| ***MATR3*** | 12 | c.G1420A | p.A474T | Male | 68.5 |
| ***MATR3*** | 12 | c.A1431C | p.Q477H | Female | 79.9 |
| ***MATR3*** | 2 | c.G755A | p.R252K | Male | 53.0 |
| ***NEK1*** | 27 | c.T2676G | p.I892M | Female | 84.3 |
| ***NEK1*** | 31 | c.3419_3422del | p.I1140Rfs*17 | Female | 60.4 |
| ***NEK1*** | 13 | c.1129_1132del | p.Q377Rfs*7 | Female | 65.3 |
| ***NEK1*** | 9 | c.C781T | p.R261C | Female | 65.6 |
| ***NEK1*** | 26 | c.2523_2526del | p.N841Kfs*53 | Female | 78.9 |
| ***NEK1*** | 15 | c.A1260C | p.E420D | Male | 72.9 |
| ***NEK1*** | 31 | c.G3373A | p.E1125K | Male | 60.6 |
| ***NEK1*** | 28 | c.C2816G | p.S939X | Female | 69.8 |
| ***NEK1*** | 30 | c.C3215T | p.T1072I | Female | 50.7 |
| ***NEK1*** | 30 | c.3214delA | p.T1072Lfs*20 | Male | 70.8 |
| ***NEK1*** | 22 | c.A1903G | p.N635D | Male | 64.4 |
| ***NEK1*** | 32 | c.C3502T | p.H1168Y | Female | 48.0 |
| ***NEK1*** | 29 | c.G2985C | p.M995I | Male | 68.6 |
| ***NEK1*** | 6 | c.C449G | p.A150G | Female | 71.0 |
| ***NEK1*** | 5 | c.G380A | p.R127Q | Female | 48.0 |
| ***NEK1*** | 15 | c.G1226A | p.W409X | Male | 61.3 |
| ***NEK1*** | 12 | c.A1074G | p.I358M | Male | 71.0 |
| ***NEK1*** | 32 | c.A3451C | p.I1151L | Male | 64.1 |
| ***NEK1*** | 4 | c.T309A | p.D103E | Female | 61.5 |
| ***NEK1*** | 18 | c.A1502G | p.N501S | Female | 52.8 |
| ***NEK1*** | 8 | c.C577T | p.L193F | Female | 65.4 |
| ***NEK1*** | 28 | c.C2816G | p.S939X | Male | 69.7 |
| ***NEK1*** | 28 | c.G2698T | p.D900Y | Female | 70.5 |
| ***NEK1*** | 2 | c.A77T | p.D26V | Female | 54.7 |
| ***NEK1*** | 29 | c.T3029C | p.I1010T | Male | 76.3 |
| ***OPTN*** | 3 | c.C265T | p.Q89X | Female | 68.8 |
| ***OPTN*** | 3 | c.C286T | p.R96C | Male | 69.3 |
| ***OPTN*** | 14 | c.C1639T | p.Q547X | Male | 61.3 |
| ***OPTN*** | 12 | c.T1499C | p.L500P | Female | 84.6 |
| ***OPTN*** | 3 | c.T263C | p.I88T | Female | 85.5 |
| ***OPTN*** | 14 | c.G1719A | p.M573I | Male | 52.7 |
| ***OPTN*** | 4 | c.G398A | p.R133K | Female | 51.5 |
| ***OPTN*** | 13 | c.C1588A | p.Q530K | Male | 78.5 |
| ***OPTN*** | 8 | c.917_921del | p.T307Sfs*3 | Male | 61.7 |
| ***OPTN*** | 3 | c.T332G | p.L111R | Female | 71.7 |
| ***OPTN*** | 3 | c.C247T | p.R83C | Female | 61.8 |
| ***OPTN*** | 6 | c.G644A | p.R215K | Female | 49.7 |
| ***OPTN*** | 12 | c.T1499C | p.L500P | Male | 75.8 |
| ***PFN1*** | 1 | c.G67A | p.V23M | Male | 73.3 |
| ***PFN1*** | 1 | c.G43C | p.G15R | Male | 48.2 |
| ***PFN1*** | 3 | c.A350G | p.E117G | Male | 63.3 |
| ***SOD1*** | 3 | c.A197G | p.N66S | Female | 65.3 |
| ***SOD1*** | 4 | c.G271A | p.D91N | Female | 70.7 |
| ***SOD1*** | 2 | c.C115G | p.L39V | Female | 46.8 |
| ***SOD1*** | 5 | c.G442A | p.G148S | Female | 69.2 |
| ***SOD1*** | 4 | c.G281A | p.G94D | Female | 57.9 |
| ***SOD1*** | 5 | c.G435C | p.L145F | Female | 44.7 |
| ***SOD1*** | 3 | c.A197G | p.N66S | Female | 48.8 |
| ***SOD1*** | 5 | c.A409T | p.K137X | Male | 44.7 |
| ***SOD1*** | 5 | c.G435C | p.L145F | Female | 50.1 |
| ***SOD1*** | 4 | c.G281A | p.G94D | Male | 37.6 |
| ***SOD1*** | 5 | c.G435T | p.L145F | Female | 61.4 |
| ***SOD1*** | 3 | c.G217A | p.G73S | Male | 48.0 |
| ***SOD1*** | 5 | c.G435C | p.L145F | Female | 66.6 |
| ***SOD1*** | 5 | c.G435C | p.L145F | Male | 60.3 |
| ***SOD1*** | 4 | c.G281A | p.G94D | Female | 68.9 |
| ***SOD1*** | 5 | c.T449C | p.I150T | Male | 52.7 |
| ***SOD1*** | 3 | c.A197G | p.N66S | Male | 72.8 |
| ***SOD1*** | 1 | c.G16A | p.V6M | Female | 78.6 |
| ***SPTLC1*** | 10 | c.T952A | p.C318S | Male | 58.2 |
| ***SPTLC1*** | 8 | c.C718T | p.R240C | Male | 52.6 |
| ***SPTLC1*** | 13 | c.G1208A | p.G403E | Female | 75.8 |
| ***SQSTM1*** | 6 | c.G775A | p.V259M | Male | 48.8 |
| ***SQSTM1*** | 7 | c.C1043T | p.P348L | Male | 53.0 |
| ***SQSTM1*** | 8 | c.C1313T | p.P438L | Female | 80.3 |
| ***SQSTM1*** | 3 | c.C447G | p.D149E | Female | 77.5 |
| ***SQSTM1*** | 6 | c.C833T | p.T278I | Male | 75.1 |
| ***SQSTM1*** | 7 | c.C1142T | p.A381V | Female | 82.4 |
| ***SQSTM1*** | 6 | c.G775C | p.V259L | Female | 77.4 |
| ***SQSTM1*** | 6 | c.G775C | p.V259L | Male | 76.6 |
| ***SQSTM1*** | 7 | c.G1084A | p.E362K | Female | 63.8 |
| ***SQSTM1*** | 8 | c.A1210G | p.M404V | Male | 67.1 |
| ***TARDBP*** | 6 | c.G1144A | p.A382T | Female | 67.0 |
| ***TARDBP*** | 6 | c.G1144A | p.A382T | Female | 59.9 |
| ***TARDBP*** | 6 | c.G1144A | p.A382T | Male | 66.6 |
| ***TARDBP*** | 6 | c.G1144A | p.A382T | Male | 70.3 |
| ***TARDBP*** | 6 | c.G1144A | p.A382T | Female | 74.4 |
| ***TARDBP*** | 6 | c.G1144A | p.A382T | Female | 78.9 |
| ***TARDBP*** | 6 | c.C1178T | p.S393L | Male | 69.6 |
| ***TARDBP*** | 6 | c.C1178T | p.S393L | Male | 73.5 |
| ***TARDBP*** | 6 | c.A800G | p.N267S | Male | 66.9 |
| ***TARDBP*** | 6 | c.G1144A | p.A382T | Male | 60.2 |
| ***TARDBP*** | 6 | c.G1144A | p.A382T | Male | 54.5 |
| ***TARDBP*** | 6 | c.G1144A | p.A382T | Female | 71.7 |
| ***TARDBP*** | 6 | c.G883A | p.G295S | Male | 66.5 |
| ***TARDBP*** | 6 | c.G1144A | p.A382T | Male | 77.2 |
| ***TBK1*** | 14 | c.G1636A | p.D546N | Male | 77.5 |
| ***TBK1*** | 11 | c.A1327T | p.I443L | Male | 61.4 |
| ***TBK1*** | 12 | c.1343_1346del | p.I450Kfs*15 | Female | 67.7 |
| ***TBK1*** | 4 | c.T254C | p.I85T | Male | 55.1 |
| ***TBK1*** | 5 | c.A521G | p.Y174C | Male | 71.8 |
| ***TBK1*** | 8 | c.C992T | p.T331I | Male | 49.9 |
| ***TBK1*** | 5 | c.A495T | p.E165D | Female | 48.6 |
| ***TBK1*** | 5 | c.G454C | p.V152L | Female | 67.0 |
| ***TBK1*** | 15 | c.A1699C | p.K567Q | Male | 62.4 |
| ***TBK1*** | 4 | c.T254C | p.I85T | Male | 77.2 |
| ***TBK1*** | 9 | c.A1179G | p.I393M | Female | 69.4 |
| ***TBK1*** | 9 | c.G1070A | p.R357Q | Male | 48.2 |
| ***TBK1*** | 11 | c.G1261A | p.V421I | Female | 78.0 |
| ***TBK1*** | 9 | c.A1179G | p.I393M | Female | 62.1 |
| ***TUBA4A*** | 2 | c.148delG | p.A50Qfs*89 | Male | 66.3 |
| ***TUBA4A*** | 4 | c.A1000T | p.T334S | Female | 62.1 |
| ***TUBA4A*** | 4 | c.G1267A | p.D423N | Male | 57.8 |
| ***TUBA4A*** | 4 | c.1184_1190del | p.G395Afs*67 | Female | 53.1 |
| ***UBQLN2*** | 1 | c.G1505A | p.G502D | Female | 45.5 |
| ***UBQLN2*** | 1 | c.C401T | p.T134I | Female | 83.4 |
| ***UBQLN2*** | 1 | c.A1172G | p.Y391C | Male | 72.3 |
| ***VAPB*** | 4 | c.C332T | p.P111L | Male | 56.9 |
| ***VCP*** | 16 | c.C2086T | p.R696C | Female | 62.3 |
| ***VCP*** | 15 | c.A2002G | p.R668G | Male | 69.4 |
| ***VCP*** | 14 | c.A1633G | p.I545V | Female | 68.8 |
| ***VCP*** | 4 | c.T265C | p.Y89H | Male | 71.3 |
| ***VCP*** | 4 | c.T265C | p.Y89H | Male | 48.4 |

**Table S4.** Complete List of Pathogenic, Likely Pathogenic, and Rare Damaging Variants Identified by Whole-Genome Sequencing in the AnswerALS Cohort.

| **Gene_name** | **Exon** | **Nucleotide** | **Aminoacid** | **Sex** | **Age At Onset** |
| --- | --- | --- | --- | --- | --- |
| **C9ORF72** | intron1 | c.-45+163GGGGCC[>24] | - | Female | 63 |
| **C9ORF72** | intron1 | c.-45+163GGGGCC[>24] | - | Male |  |
| **C9ORF72** | intron1 | c.-45+163GGGGCC[>24] | - | Female | 63 |
| **C9ORF72** | intron1 | c.-45+163GGGGCC[>24] | - | Female | 60 |
| **C9ORF72** | intron1 | c.-45+163GGGGCC[>24] | - | Male | 50 |
| **C9ORF72** | intron1 | c.-45+163GGGGCC[>24] | - | Male | 64 |
| **C9ORF72** | intron1 | c.-45+163GGGGCC[>24] | - | Female | 61 |
| **C9ORF72** | intron1 | c.-45+163GGGGCC[>24] | - | Female | 55 |
| **C9ORF72** | intron1 | c.-45+163GGGGCC[>24] | - | Male | 54 |
| **C9ORF72** | intron1 | c.-45+163GGGGCC[>24] | - | Male | 48 |
| **C9ORF72** | intron1 | c.-45+163GGGGCC[>24] | - | Female | 64 |
| **C9ORF72** | intron1 | c.-45+163GGGGCC[>24] | - | Female | 59 |
| **C9ORF72** | intron1 | c.-45+163GGGGCC[>24] | - | Male | 58 |
| **C9ORF72** | intron1 | c.-45+163GGGGCC[>24] | - | Male | 50 |
| **C9ORF72** | intron1 | c.-45+163GGGGCC[>24] | - | Female | 57 |
| **C9ORF72** | intron1 | c.-45+163GGGGCC[>24] | - | Female | 66 |
| **C9ORF72** | intron1 | c.-45+163GGGGCC[>24] | - | Male | 64 |
| **C9ORF72** | intron1 | c.-45+163GGGGCC[>24] | - | Female | 61 |
| **C9ORF72** | intron1 | c.-45+163GGGGCC[>24] | - | Female | 58 |
| **C9ORF72** | intron1 | c.-45+163GGGGCC[>24] | - | Male | 47 |
| **C9ORF72** | intron1 | c.-45+163GGGGCC[>24] | - | Female | 60 |
| **C9ORF72** | intron1 | c.-45+163GGGGCC[>24] | - | Male | 62 |
| **C9ORF72** | intron1 | c.-45+163GGGGCC[>24] | - | Female | 53 |
| **C9ORF72** | intron1 | c.-45+163GGGGCC[>24] | - | Male | 53 |
| **C9ORF72** | intron1 | c.-45+163GGGGCC[>24] | - | Male | 57 |
| **C9ORF72** | intron1 | c.-45+163GGGGCC[>24] | - | Female | 55 |
| **C9ORF72** | intron1 | c.-45+163GGGGCC[>24] | - | Female | 70 |
| **C9ORF72** | intron1 | c.-45+163GGGGCC[>24] | - | Female | 40 |
| **C9ORF72** | intron1 | c.-45+163GGGGCC[>24] | - | Female | 51 |
| **C9ORF72** | intron1 | c.-45+163GGGGCC[>24] | - | Male | 50 |
| **C9ORF72** | intron1 | c.-45+163GGGGCC[>24] | - | Female | 50 |
| **C9ORF72** | intron1 | c.-45+163GGGGCC[>24] | - | Female | 68 |
| **C9ORF72** | intron1 | c.-45+163GGGGCC[>24] | - | Female | 56 |
| **C9ORF72** | intron1 | c.-45+163GGGGCC[>24] | - | Male | 60 |
| **C9ORF72** | intron1 | c.-45+163GGGGCC[>24] | - | Male | 56 |
| **C9ORF72** | intron1 | c.-45+163GGGGCC[>24] | - | Male | 52 |
| **C9ORF72** | intron1 | c.-45+163GGGGCC[>24] | - | Female | 51 |
| **C9ORF72** | intron1 | c.-45+163GGGGCC[>24] | - | Female | 69 |
| **C9ORF72** | intron1 | c.-45+163GGGGCC[>24] | - | Female | 79 |
| **C9ORF72** | intron1 | c.-45+163GGGGCC[>24] | - | Male | 69 |
| **C9ORF72** | intron1 | c.-45+163GGGGCC[>24] | - | Female | 69 |
| **C9ORF72** | intron1 | c.-45+163GGGGCC[>24] | - | Male | 58 |
| **CHMP2B** | 2 | c.G270A | p.R19Q | Female | 69 |
| **CHMP2B** | 3 | c.G420A | p.R69Q | Female | 61 |
| **FUS** | 15 | c.G1648C | p.R524S | Female | 27 |
| **FUS** | 14 | c.1472delT | p.V467X | Female | 61 |
| **KIF5A** | 1 | c.C595T | p.Q123X | Male | 47 |
| **KIF5A** | 1 | c.546_547delinsTGGG | p.G183WfsX? | Male | 67 |
| **KIF5A** | 12 | c.G1378C | p.G384R | Male | 64 |
| **KIF5A** | 25 | c.G3109A | p.A961T | Female | 70 |
| **KIF5A** | 26 | c.A3218T | p.N997I | Male | 58 |
| **NEK1** | 31 | c.G3664A | p.R1044X | Male | 75 |
| **NEK1** | 31 | c.G3641C | p.S1036X | Female | 65 |
| **NEK1** | 31 | c.TA3582T | p.H1016X | Female | 65 |
| **NEK1** | 24 | c.C2671T | p.V713M | Male | 62 |
| **NEK1** | 24 | c.C2671T | p.V713M | Male | 55 |
| **NEK1** | 24 | c.C2671T | p.V713M | Male | 65 |
| **NEK1** | 24 | c.C2671T | p.V713M | Male | 40 |
| **NEK1** | 32 | c.GT3875G | p.N1114X | Male | 50 |
| **NEK1** | 24 | c.C2671T | p.V713M | Female | 29 |
| **NEK1** | 17 | c.G1888A | p.Q452X | Male | 56 |
| **OPTN** | 5 | c.684_685delinsAG | p.K229RfsX? | Female | 54 |
| **OPTN** | 6 | c.GA869G | p.E189X | Female | 59 |
| **OPTN** | 8 | c.C1088A | p.S262X | Male | 44 |
| **OPTN** | 15 | c.G1937A | p.R545Q | Male | 47 |
| **PFN1** | 3 | c.T486C | p.E117G | Female | 54 |
| **PFN1** | 3 | c.T486C | p.E117G | Male | 40 |
| **PFN1** | 2 | c.C106T | p.R36X | Male | 47 |
| **PFN1** | 2 | c.G148A | p.R36X | Male | 58 |
| **PFN1** | 2 | c.A347C | p.C71G | Female | 52 |
| **SOD1** | 1 | c.C91T | p.A5V | Female | 57 |
| **SOD1** | 1 | c.C91T | p.A5V | Male | 44 |
| **SOD1** | 4 | c.T418C | p.I114T | Female | 39 |
| **SOD1** | 4 | c.T418C | p.I114T | Male | 63 |
| **SOD1** | 1 | c.200delC | p.V41X | Male | 56 |
| **SOD1** | 1 | c.200delC | p.V41X | Male | 54 |
| **SOD1** | 5 | c.A451G | p.D125G | Female | 59 |
| **SOD1** | 4 | c.T418C | p.I114T | Female | 39 |
| **SOD1** | 1 | c.C91T | p.A5V | Female | 27 |
| **SOD1** | 4 | c.T340C | p.V88A | Male | 67 |
| **SOD1** | 3 | c.T270C | p.F65L | Male | 41 |
| **SOD1** | 1 | c.G90A | p.A5T | Male | 46 |
| **SOD1** | 2 | c.A223G | p.H49R | Female | 60 |
| **SOD1** | 4 | c.A349C | p.D91A | Female | 51 |
| **SOD1** | 3 | c.T270C | p.F65L | Female | 53 |
| **SOD1** | 4 | c.G358C | p.G94A | Male | 57 |
| **SOD1** | 4 | c.A337G | p.N87S | Male | 47 |
| **SOD1** | 4 | c.C346T | p.A90V | Male | 57 |
| **SOD1** | 4 | c.G378A | p.E101K | Male | 46 |
| **SPTLC1** | 4 | c.C347T | p.W108X | Male | 61 |
| **SQSTM1** | 8 | c.C1207T | p.P392L | Female | 60 |
| **SQSTM1** | 8 | c.C1207T | p.P392L | Female | 49 |
| **SQSTM1** | 1 | c.C130T | p.A33V | Male | 65 |
| **SQSTM1** | 8 | c.C1207T | p.P392L | Male | 45 |
| **SQSTM1** | 1 | c.C130T | p.A33V | Male | 59 |
| **SQSTM1** | 7 | c.C1192T | p.P387L | Male | 56 |
| **TARDBP** | 6 | c.G1145T | p.G348V | Male | 41 |
| **TARDBP** | 6 | c.A1270G | p.N390D | Male | 25 |
| **TARDBP** | 4 | c.C400T | p.L134X | Male | 65 |
| **TARDBP** | 6 | c.A1270G | p.N390D | Male | 66 |
| **TARDBP** | 6 | c.A1111G | p.M337V | Female | 52 |
| **TARDBP** | 6 | c.G1252C | p.G384R | Female | 63 |
| **TBK1** | 18 | c.T2040A | p.Y647* | Female | 50 |
| **TBK1** | 8 | c.C928G | p.L277V | Male | 71 |
| **UBQLN2** | 1 | c.C1737A | p.P497H | Female | 29 |
| **UBQLN2** | 1 | c.C1820T | p.P525S | Male | 45 |
| **VCP** | 5 | c.C736T | p.R155H | Male | 45 |
